# Impact of pulmonary complications following esophagectomy on long-term survival: multivariate meta-analysis and restricted mean survival time assessment

**DOI:** 10.1007/s13304-024-01761-2

**Published:** 2024-02-06

**Authors:** Michele Manara, Davide Bona, Luigi Bonavina, Alberto Aiolfi, OGSA Group for Esophagogastric Surgery, Gianluca Bonitta, Juxhin Guraj, Guglielmo Guerrazzi, Giampiero Campanelli, Marta Cavalli, Călin Popa, Diana Schlanger, Ewen A Griffiths, Antonio Biondi

**Affiliations:** 1 Division of General Surgery, Department of Biomedical Science for Health, I.R.C.C.S. Ospedale Galeazzi-Sant’Ambrogio, University of Milan, Via C. Belgioioso N. 173, 20151 Milan, Italy; 2 Division of General Surgery, Department of Biomedical Science for Health, I.R.C.C.S. Policlinico San Donato, University of Milan, Milan, Italy

**Keywords:** Esophagectomy, Postoperative complications, Esophageal cancer, Pulmonary complications, Long-term survival

## Abstract

Pulmonary complications (PC) are common after esophagectomy and their impact on long-term survival is not defined yet. The present study aimed to assess the effect of postoperative PCs on long-term survival after esophagectomy for cancer. Systematic review of the literature through February 1, 2023, was performed. The included studies evaluated the effect of PC on long-term survival. Primary outcome was long-term overall survival (OS). Cancer-specific survival (CSS) and disease-free survival (DFS) were secondary outcomes. Restricted mean survival time difference (RMSTD), hazard ratio (HR), and 95% confidence intervals (CI) were used as pooled effect size measures. Eleven studies were included (3423 patients). Overall, 674 (19.7%) patients developed PC. The RMSTD analysis shows that at 60-month follow-up, patients not experiencing PC live an average of 8.5 (95% CI 6.2–10.8; *p* < 0.001) months longer compared with those with PC. Similarly, patients not experiencing postoperative PC seem to have significantly longer CSS (8 months; 95% CI 3.7–12.3; *p* < 0.001) and DFS (5.4 months; 95% CI 1.6–9.1; *p* = 0.005). The time-dependent HRs analysis shows a reduced mortality hazard in patients without PC at 12 (HR 0.6, 95% CI 0.51–0.69), 24 (HR 0.64, 95% CI 0.55–0.73), 36 (HR 0.67, 95% CI 0.55–0.79), and 60 months (HR 0.69, 95% CI 0.51–0.89). This study suggests a moderate clinical impact of PC on long-term OS, CSS, and DFS after esophagectomy. Patients not experiencing PC seem to have a significantly reduced mortality hazard up to 5 years of follow-up.

**Graphical abstract:**

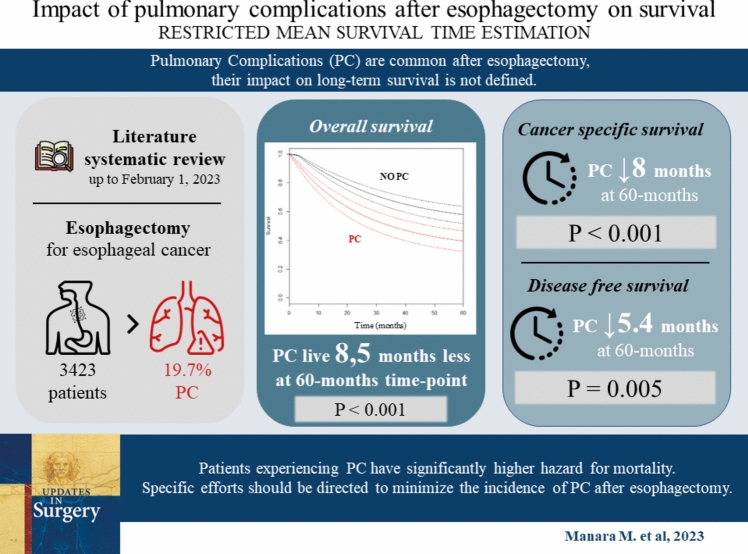

**Supplementary Information:**

The online version contains supplementary material available at 10.1007/s13304-024-01761-2.

## Introduction

Esophageal cancer is the eight most diagnosed cancer and sixth leading cause of cancer-related mortality worldwide [1]. Long-term mortality rates are dismal despite multimodal treatment, with 5-year relative survival rates ranging between 46% in localized disease to 5% in advanced disease [2]. Esophagectomy remains the most important component of curative treatment, but postoperative morbidity and mortality rates are still high despite the significant efforts to minimize complications [3–11].

Pulmonary complications (PC), including pleural effusions, atelectasis, pneumonia, pulmonary embolism, and respiratory failure, are frequently described with an estimated incidence up to 30% [12, 13]. These complications are associated with prolonged hospital stay, increased cost of care, need for additional treatments, and substantial peri-operative mortality. The impact of PC on long-term survival is not defined with previous series reporting conflicting results on long-term survival [14–27].

The aim of the present meta-analysis was to assess the effect of postoperative PC on long-term survival after esophagectomy for cancer.

## Materials and methods

A systematic review was designed according to the Preferred Reporting Items for Systematic Review and Meta-Analyses (PRISMA) guidelines [28]. A literature review of PubMed, MEDLINE, Scopus, Web of Science, Cochrane Central Library, and ClinicalTrials.gov was performed [29], using the following Medical Subject Heading (MeSH) search terms: esophageal cancer, esophageal neoplasm, pulmonary complication, pneumonia, atelectasis, respiratory failure, pulmonary embolism, pleural effusion, complication, postoperative compl*, survival, overall surv*, cancer specific survival. Multiple combinations of search terms were used. Articles published from January 1, 2000, to February 1, 2023, were screened, together with relevant articles’ references. This study is based on previously published studies and, therefore, did not require any additional ethical approval.

### Eligibility criteria

Studies reporting data on overall survival (OS), cancer specific survival (CSS), and disease free survival (DFS) of patients undergoing esophagectomy for cancer were considered eligible for inclusion. All studies that reported Kaplan–Meier long-term survival curves comparing PC and no PC were included. Studies reporting mixed population data and lacking PC comparative analysis or outcome assessment, were excluded. Editorials, review articles, case reports and studies involving small numbers of patients (≤ 20 cases) were excluded. Studies reporting similar cohorts of patients and overlapping populations were identified and those with broader inclusion criteria were considered.

### Selection process

The literature review was performed separately by three independent reviewers (MM, GG, and JG) according to the established inclusion criteria. Screening by title and abstract was implemented with Rayyan Intelligent Systematic Review, and if the inclusion criteria were met, the entire article was reviewed. After duplicates were removed, disagreements were resolved by two additional blinded reviewers (AA and DB).

### Data collection process

Data were analyzed and registered separately by reviewers (MM, GG, and JG) filling out pro forma tables on Google Sheets with predetermined variables. The variables included in the study were author, publication year, country, inclusion criteria, exclusion criteria, study design, population demographics (number, age, sex, body mass index, American Society of Anesthesiologists physical status, number of PC), tumor characteristics (histology, location, neoadjuvant and adjuvant therapy), and surgical treatment (surgical approach, anastomotic technique, lymphadenectomy fields, pathologic tumor staging, and residual tumor classification). Kaplan–Meier curves regarding the outcomes of interest were collected along with these data. All data were compared at the end of the review process by two other authors (AA and GB) to determine and resolve discrepancies.

### Outcome of interest and definition

Primary outcome was OS while CSS and DSF were secondary outcomes. OS was defined as the time from surgery to the last known follow-up and death. CSS was defined as the duration from the date of diagnosis until death due to esophageal cancer other than other causes. DFS was defined as the time from surgical resection to local recurrence. Survival data were extracted using Kaplan–Meier survival curves. PC were defined as the presence of one or more of the following postoperative conditions: initial ventilatory support for more than 48 h or reintubation for respiratory failure, pneumonia requiring additional medical treatments, acute respiratory distress syndrome, or any medical event affecting the lung parenchyma requiring intervention or surgical treatment [14, 19].

### Quality assessment

The methodological quality of the included studies was independently assessed by three authors (MM, AA, and GB) using the ROBINS-I tool for observational studies [30]. The following domains included: confounding bias, selection bias, classification bias, intervention bias, missing data bias, outcomes measurement bias, and reporting bias. Each domain is evaluated with “Low”, “Moderate”, “Serious”, or “Critical”. The categories of judgment for each study are low, moderate, serious, and critical risk of bias.

### Statistical analysis

The results of the systematic review were summarized qualitatively into a frequentist meta-analysis of restricted mean survival time difference (RMSTD) [31–33] Individual patient time-to-event data were reconstructed from Kaplan–Meier curves according to Guyot [34]. The Kaplan–Meier curves were digitalized using Get Data Graph Digitizer software (http://getdata-graph-digitizer.com). The calculation of pooled (RMSTD) was performed using a random effect multivariate meta-analysis borrowing strength across time points with a within-trial covariance matrix derived by bootstrapping with 1,000 iterations; the restriction time was 60 months. In addition, using IPD, we performed a flexible hazard-based regression model with the inclusion of a normally distributed random intercept. In the periocular, we modeled the baseline hazard described by the exponential of a B-spline of degree 3 with no interior knots, and the model selection was driven according to the Akaike Information Criterion (AIC). The time-dependent effects of surgical treatment were parameterized as interaction terms between surgical treatment and baseline hazard and statistically tested using the likelihood ratio test. The hazard function plot was performed using marginal prediction [35]. Two-sided *p* values were considered statistically significant when less than 0.05 and the CIs were computed at 95%. All analyses were carried out using the R software application (version 3.2.2; R Foundation, Vienna, Austria) [36].

## Results

### Systematic review

**Fig. 1:**
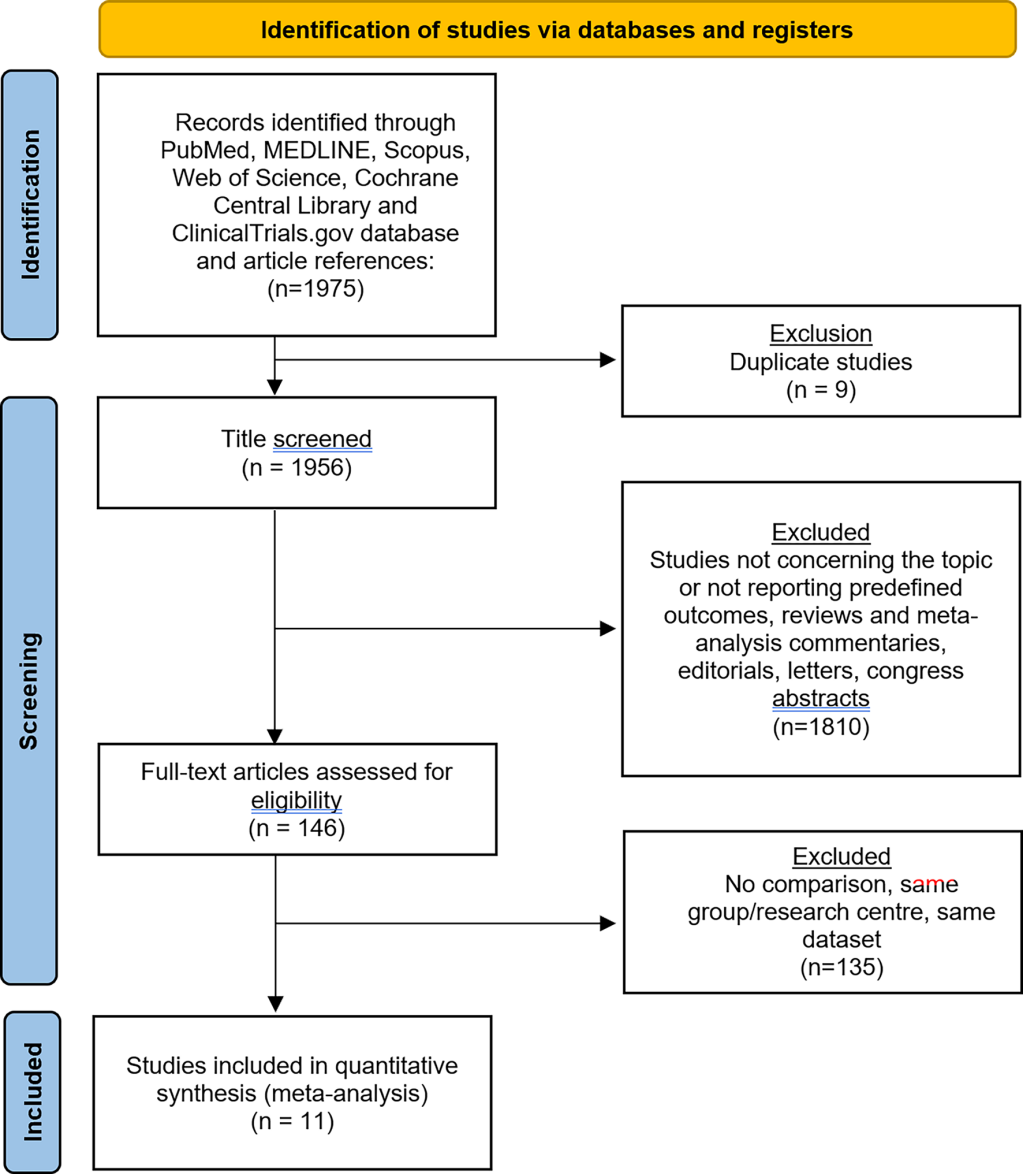
The Preferred Reporting Items for Systematic Reviews checklist (PRISMA) diagram

The flowchart of the selection process is shown in Fig. 1. Overall, 1956 publications were screened after duplicate removal, and 146 were identified for full-text review. After evaluation, 11 observational studies met the inclusion and exclusion criteria and were included in the quantitative analysis. The quality of the included studies is listed in Supplementary Table 1.

Quantitative analysis included 3423 patients undergoing esophagectomy for cancer in high-volume centers (Table 1). Postoperative PC were reported in 674 (19.7%) patients. The age of the patient population ranged from 35 to 85 years, and 86.8% were males. The American Society of Anesthesiologists score was reported in four studies [17, 18, 22, 25], the body mass index in two studies [22, 25], while the Charlson comorbidity index was not reported in any study. History of tobacco smoking and information on preoperative pulmonary function was reported in five [16, 17, 19, 22, 24] and four studies, respectively [16, 23–25]. Squamous cell carcinoma was the most frequently reported tumor histology (90%) followed by adenocarcinoma (8%). Pathological tumor staging according to the sixth, seventh, and eighth editions of the American Joint Committee on Cancer was detailed in nine studies; stage 0–I, 33.6%; stage II, 24.7%; stage III, 34.3%; and stage IV, 7.4%. The tumor location was reported in eight studies with distribution in upper (11.6%), medium (55.8%), and lower (32.6%) esophagus [14, 17, 20–25]. Neoadjuvant chemoradiation treatment was reported 56% of patients with different protocols and chemotherapy regimens (PF vs. FAP vs. DCF). Adjuvant treatment was specified in two studies, in 196/786 (24.9%) patients. Open, hybrid, and totally minimally invasive Ivor-Lewis or McKeown esophagectomy were mainly described depending on operating surgeon discretion and tumor location. Lymph node dissection was extended to 2 or 3 fields according to clinical preoperative staging. The anastomotic technique varied among the included studies in terms of both the location and route of reconstruction according to tumor location and operating surgeon preferences.

**Table 1:** Demographic, clinical, and operative data for patients undergoing esophagectomy for cancer

References	Country	Study design	No. pts	Sex M	Age (years)	Tumor histology (SCC-ADK-Other)	Location (U-M-L)	Neoadjuvant-adjuvant treatment	pStage 0–I	pStage II	pStage III	pStage IV	Surgical approach
Kinugasa et al. [16]	Japan	Ret	118	109	63.2 ± 8.5	118-0-0	0-118-0	0-nr	26	37	33	22	Open
D'annoville et al. [14]	France	Ret	384	286	60.1 ± 10	127-214-0	14-77-250	179-nr	nr	nr	nr	nr	Open
Booka et al. [17]	Japan	Ret	284	256	nr	255-19-10	40-140-104	92–63	86	73	85	13	Open-Hyb-MIE
Yamashita et al. [18]	Japan	Ret	255	220	65 (35–85)	255-0-0	nr	255-nr	49	76	120	10	Open
Baba et al. [19]	Japan	Ret	502	445	65.7 ± 9	502-0-0	nr	202–133	225	85	156	36	Open-Hyb-MIE
Saeki et al. [20]	Japan	Ret	580	504	nr	580-0-0	102-292-186	285-nr	nr	nr	nr	nr	Open
Kataoka et al. [21]	Japan	Ret	152	133	61 (38–75)	151-0-1	12-78-62	152-nr	0	78	74	0	Open-Hyb-MIE
Hayami et al. [22]	Japan	Ret	70	65	64.6 ± 7.9	70-0-0	28-29-13	70-nr	23	15	31	1	nr
Fujishima et al. [23]	Japan	Ret	123	104	nr	123-0-0	15-45-63	63-nr	29	33	54	7	Open-Hyb-MIE
Tanaka et al. [24]	Japan	Ret	484	431	66.8 ± 8.5	484-0-0	93-236-155	448-nr	167	118	146	53	Open-Hyb-MIE
Yoshida et al. [25]	Japan	Ret	471	419	66 ± 8	420-41-10	2-448-21	190-nr	211	85	136	39	Open-Hyb-MIE

Data are reported as numbers, mean ± standard deviation, and median (range)

*Ret* retrospective, *SCC* squamous cell carcinoma, *ADK* adenocarcinoma, *U* upper esophagus, *M* medium esophagus, *L* lower esophagus, *pStage* pathologic tumor stage, reported according to the to the 6th, 7th, and 8th edition of the American Joint Committee on Cancer (AJCC), *Hyb* hybrid esophagectomy, *MIE* minimally invasive esophagectomy, *nr* not reported

### Primary outcome: OS

**Fig. 2:**
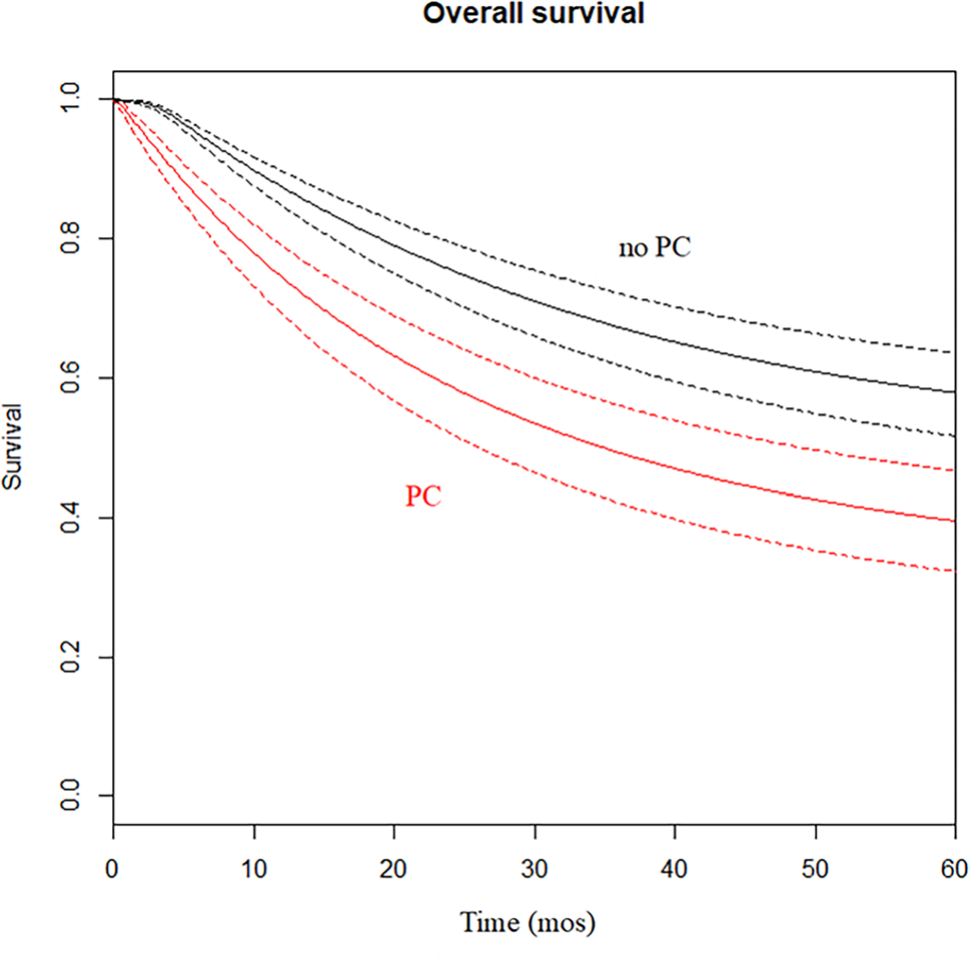
Estimated pooled overall survival (*Y* axis) for patients with pulmonary complication (red line) and without pulmonary complication (black line). Time (*X* axis) expressed in months. Relative 95% CI in dashed tract. PC Pulmonary Complications; mos months (Color figure online)

The clinical estimation of RMSTD was calculated from 11 studies reporting Kaplan–Meier curves [16, 17, 19–25]. All included studies had a minimum follow-up of 5 years. The RMSTD and time horizons for the OS are presented in Table 2 and graphically presented in Fig. 2. Multivariate meta-analysis with analytically derived covariance resulted in a combined RMSTD estimate of 1 month at 12-month time point (95% CI 0.5–1.4), meaning that patients not experiencing PC live 1 month longer than those experiencing PC. This result is statistically significant (*p* < 0.001). At *τ*_2_ = 24-month follow-up, the combined effect from the multivariate meta-analysis with analytically derived covariance is 2.7 months (95% CI 1.9–3.5; *p* < 0.001). At *τ*_3_ = 36-month, the combined effect from the multivariate meta-analysis with analytically derived covariance is 4.5 months (95% CI 3.4–5.7; *p* < 0.001). At *τ*_4_ = 48-month, the combined effect from the multivariate meta-analysis with analytically derived covariance is 6.7 months (95% CI 5.2–8.2; *p* < 0.001). Finally, at *τ*_5_ = 60-month, the combined effect from the multivariate meta-analysis with analytically derived covariance is 8.5 months (95% CI 6.2–10.8; *p* < 0.001).

**Table 2:** The restricted mean survival time difference (RMSTD) for overall survival restricted to 60 months at different time horizons for the no pulmonary complication vs. pulmonary complication comparison

Time horizon	No. trials	RMSTD (mos)	SE	95% CI	*p* value
6-month	9	0.3	0.1	0.05–0.5	0.002
12-month	9	1.0	0.2	0.5–1.4	< 0.001
24-month	9	2.7	0.4	1.9–3.5	< 0.001
36-month	9	4.5	0.6	3.4–5.7	< 0.001
48-month	9	6.7	0.8	5.2–8.2	< 0.001
60-month	9	8.5	1.2	6.2–10.8	< 0.001

*SE* standard error, *95% CI* confidence intervals, *mos* months

**Fig. 3:**
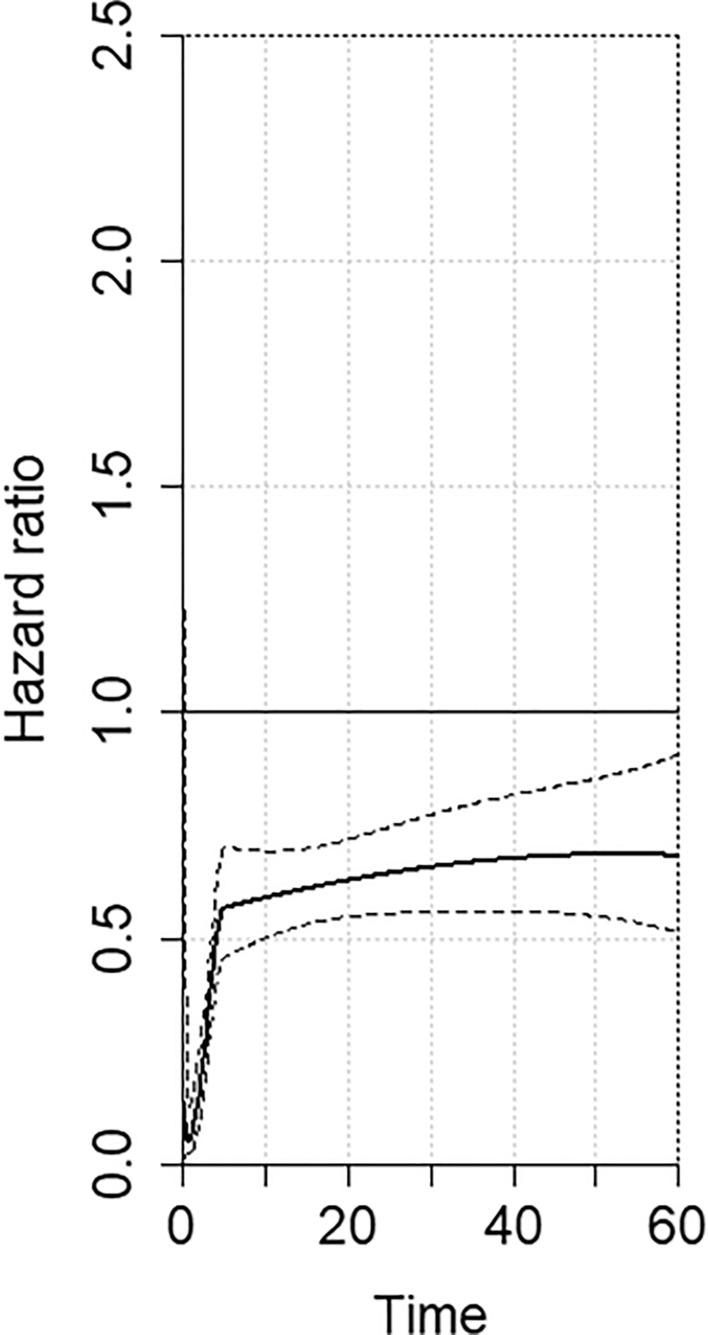
No pulmonary complication vs. pulmonary complication overtime hazard ratio variations (*Y* axis). Continued tracts represent the estimated pooled hazards while dotted tracts represent the 95% Confidence Interval (95% CI). Time (*X* axis) expressed in months

Considering the non-proportional hazard model (*p* < 0.001), the time-varying hazard ratios for no PC versus PC are shown in Fig. 3. Specifically, no PC is associated with a significantly estimated lower hazard for mortality at 12 months (HR 0.60, 95% CI 0.51–0.69), 24 months (HR 0.64, 95% CI 0.55–0.73), 36 months (HR 0.67, 95% CI 0.55–0.79), 48 months (HR 0.68, 95% CI 0.55–0.84), and 60 months (HR 0.68, 95% CI 0.51–0.89) compared to PC (Table 3).

**Table 3:** Time-dependent hazard ratio analysis for overall survival in the comparison no pulmonary complication vs. pulmonary complication

Time horizon	No PC vs. PC HR (95% CI)
12-month	0.60 (0.51–0.69)
24-month	0.64 (0.55–0.73)
36-month	0.67 (0.55–0.79)
48-month	0.68 (0.55–0.84)
60-month	0.68 (0.51–0.89)

*PC* pulmonary complications, *95% CI* confidence intervals

### Secondary outcomes: CSS/DFS

RMSTD and time horizons for CSS and DFS are detailed in Supplementary Tables 2 and 3. Specifically, multivariate meta-analysis resulted in a combined RMSTD for CSS estimate of 8 months at 60-month time point (95% CI 3.7–12.3), meaning that patients not experiencing PC live 8 months longer than those experiencing PC (*p* < 0.001). Similarly, multivariate meta-analysis resulted in a combined RMSTD for DFS estimate of 5.4 months at 60-month time point (95% CI 1.6–9.1), meaning that patients not experiencing PC live 5.4 months longer than those experiencing PC (*p* = 0.005).

## Discussion

The present study shows that PC after esophagectomy has a moderate clinical impact on long-term OS, CSS and DFS. Furthermore, the time-dependent HR analysis shows that patients not experiencing PC seem to have a significantly lower mortality hazard up to 60 months follow-up.

Despite recent improvements in surgical techniques and perioperative management, esophagectomy remains associated with high morbidity and mortality rates. The detrimental effect of postoperative PC on short-term outcomes has already been described by some authors and were associated with augmented costs, reduced quality of life, increased risk for re-intubation and perioperative mortality [6, 37, 38]. However, the specific burden of PC on long-term outcomes and survival is still debated. In our study, we observed that PC has a moderate impact on long-term OS, CSS, and DFS. Two principal factors may theoretically explain these results. First, postoperative infectious complications have been reported to be associated with increased concentration of inflammatory interleukins (IL-6, IL-8) [39–41]. This may be associated with tumor progression, residual cancer cells stimulation, and tumor metastasis facilitation [42, 43]. Second, general condition’s worsening induced by PC may determine delay or poor tolerance to additional adjuvant treatments affecting cancer-related mortality. This mechanism may also affect cancer-unrelated mortality, especially in patients with deteriorated preoperative pulmonary function, comorbidities, or heavy smokers [21, 41, 44–46]. We can also assume that fragile patients or heavy smokers could be more prone to experiencing PC and less likely to survive in the long term. In our study, we found significantly reduced 5-year OS in patients that experienced PC. This finding is consistent with Kinugasa et al., who described reduced 5-year OS in patients experiencing PC after esophagectomy (HR 2.37, *p* = 0.018) [16]. Also, Booka et al. observed a significantly reduced 5-year OS in patients experiencing PC (40.6% vs. 52.3%, *p* = 0.035) [17]. Similarly, Baba et al. reported a detrimental impact of PC on OS (HR 1.6, *p* = 0.029) [19] while the JCOG9907 trial showed higher hazard for mortality in patients with PC (HR 1.52, *p* = 0.048) [21]. In contrast, a recent dataset-based analysis from the EsoBenchmark database showed no association between PC and reduced long-term survival in the context of minimally invasive esophagectomy [47]. In our study, we found that PC seem to significantly impact 5-year CSS and DFS. This is similar to Yamashita et al. (HR 2.5, *p* = 0.007) and Baba et al. (*p* = 0.0062) that observed a statistically significant detrimental effect of PCs on CSS [18, 19]. Differently, Kinugasa et al. reported no significant implications of pneumonia in CSS (*p* = 0.22) [16]. Related to DFS, D’Annoville et al. and Kataoka and colleagues did not describe a significant effect of PCs on DFS [14, 21]. Conversely, Tanaka et al. observed a negative prognostic impact of pneumonia on DFS (*p* = 0.0365) [24].

After RMSTD analysis, we evaluated the survival data and HRs extrapolated from survival curves on OS. Usually, HRs are used to estimate the treatment effect for time-to-event endpoints and provide an estimate of the ratio of the hazard rates between the experimental and control groups over the entire study duration. A previous meta-analysis proposed HR analysis by reporting a single calculation that was presumed constant over the entire duration of the study [26]. However, HRs are time-dependent variables, change over time and are useful to describe the magnitude and direction of survival outcomes [48]. As expected, the analysis of risk-time variations showed that the hazard of mortality changes in relation to postoperative follow-up with significantly reduced hazard for mortality in patients that did not experienced PCs up to 5-year follow-up. Therefore, we can hypothetically assume that PC have a negative prognostic impact on OS up to 5 years after esophagectomy.

Three principal issues should be considered while interpreting our results. First, the effect of centralization in high volume hospitals has been reported to be associated with reduced risk of PC [49–53]. Since all included studies were accomplished in high-volume referral centers, our results may represent the best possible scenario and might not be generalizable. Second, surgical approach has been shown to significantly impact postoperative PC [4, 54–58]. Our study included aggregated and heterogeneous surgical approaches for esophagectomy (open vs. hybrid vs. totally MIE) therefore a specific stratification was not feasible. Third, intra- and perioperative multidisciplinary management, including pre-habilitation, and combination with protective lung ventilation protocols, have been shown to minimize risk of postoperative PC [59–62]. In our study, no clear data were available regarding patient management protocols, therefore, no specific inferences or sub analysis could be pursued.

The major strength of the present meta-analysis is the evaluation of long-term survival between PC and non-PC using HR and RMSTD. RMSTD has gained increasing acceptance in oncology as it is a powerful, robust, and interpretable tool for assessing the clinical survival benefit of a specific treatment over another. It matches the area under the survival curves and is easier to interpret than HR and RR, which may be misinterpreted because both assume constant risk during follow-up.

This study has some limitations that need to be considered. There was patient baseline heterogeneity (i.e., demographics, comorbidities, nutritional status, smoking history, etc.). Preoperative data regarding pulmonary function (i.e., smoking cessation, respiratory rehabilitation, preoperative spirometry), oncologic data (i.e., staging, histology, grading, neoadjuvant or adjuvant treatment, extent of lymphadenectomy), and specific information on multidisciplinary perioperative care teams or enhanced recovery after surgery programs were heterogeneous and puzzled [63]. We included studies published in a time range of almost 20 years, during which oncological protocols have varied significantly with a possible effect on pulmonary complications and survival. Our results may not be generalized, because the sample was predominantly from Eastern countries with a possible influence in tumor epidemiology and genomic characterization [64]. Different surgical procedures have been incorporated in terms of anastomosis location (thoracic vs. cervical) and techniques with no clear data correction for early (30-day vs. 90-day) mortality [65]. Finally, various definitions of PC were adopted among included studies.

## Conclusion

This study suggests a moderate clinical impact of PC on long-term OS, CSS, and DFS after esophagectomy. Patients experiencing PC have significantly higher hazard for mortality compared to patients that did not experience PC. Specific efforts should be adopted to optimize preoperative assessment, intraoperative management, and peri-surgical management to possibly minimize their incidence.

### Supplementary Information

Below is the link to the electronic supplementary material.

Supplementary file1 (DOCX 42 kb)

## Data Availability

The data collected and analyzed during the current review are available from the corresponding author on reasonable request.

## Declarations

### Registration

This systematic review and meta-analysis is registered with PROSPERO. Registration number CRD42023428603.
