# Supplementary material for: Impact of pulmonary complications following esophagectomy on long-term survival: multivariate meta-analysis and restricted mean survival time assessment
Source: Updates Surg. 2024 Feb 6;76(3):757–67. doi: 10.1007/s13304-024-01761-2 (PMC11129973; doi:10.1007/s13304-024-01761-2)
Supplement: Supplementary file 1 — Supplementary file1 (DOCX 42 kb) [file 13304_2024_1761_MOESM1_ESM.docx]

**Supplementary Table 1.** Quality assessment of the included studies (ROBINS-I tool).

| **Study** | **Confounding Bias** | **Selection Bias** | **Classification Bias** | **Intervention Bias** | **Missing Data Bias** | **Measurement Bias** | **Reporting Bias** | **Bias** |
| --- | --- | --- | --- | --- | --- | --- | --- | --- |
| Kinugasa et al. 2004^15^ | Seriuos | Moderate | Serious | Moderate | NI | Moderate | Moderate | Moderate |
| D'annoville et al. 2012^13^ | Serious | Serious | Moderate | Moderate | NI | Moderate | Serious | Serious |
| Booka et al. 2015^16^ | Moderate | Serious | Moderate | Moderate | Low | Moderate | Moderate | Moderate |
| Yamashita et al. 2016^17^ | Moderate | Serious | Moderate | Moderate | Low | Moderate | Moderate | Moderate |
| Baba et al, 2016^18^ | Serious | Moderate | Moderate | Moderate | Moderate | Moderate | Moderate | Moderate |
| Saeki et al, 2017^19^ | Serious | Serious | Moderate | Moderate | Serious | Moderate | Moderate | Moderate |
| Kataoka et al. 2017^20^ | Low | Low | Moderate | Low | Moderate | Moderate | Moderate | Moderate |
| Hayami et al. 2017^21^ | Serious | Serious | Moderate | Moderate | Moderate | Moderate | Moderate | Moderate |
| Fujishima et al. 2020^22^ | Moderate | Moderate | Moderate | Moderate | Low | Moderate | Moderate | Moderate |
| Tanaka et al, 2021^23^ | Serious | Serious | Moderate | Moderate | Moderate | Moderate | Moderate | Moderate |
| Yoshida et al, 2022^24^ | Serious | Moderate | Moderate | Moderate | Low | Moderate | Moderate | Moderate |

**Legend 1.** Each domain is evaluated with one of the following: Low, Moderate, Serious, Critical, NI (No Information). The categories of judgement for each study are low, moderate, serious, and critical risk of bias.

**Supplementary table 2.** The restricted mean survival time difference (RMSTD) for Cancer Specific Survival restricted to 60 months at different time horizons for the no pulmonary complication vs. pulmonary complication comparison.

| **Time Horizon** | **No. Trials** | **RMSTD (mos)** | **SE** | **95% CI** | ***p value*** |
| --- | --- | --- | --- | --- | --- |
| 6-month | 3 | 0.2 | 0.05 | 0.1-0.3 | <0.001 |
| 12-month | 3 | 0.9 | 0.4 | 0.2-1.7 | 0.015 |
| 24-month | 3 | 2.5 | 0.8 | 0.9-4.1 | 0.002 |
| 36-month | 3 | 4.2 | 1.1 | 2-6.4 | <0.001 |
| 48-month | 3 | 6.1 | 1.3 | 3.6-8.7 | <0.001 |
| 60-month | 3 | 8 | 2.2 | 3.7-12.3 | <0.001 |

**Legend 2.** SE standard error; 95% CI confidence intervals; mos months.

**Supplementary table 3.** The restricted mean survival time difference (RMSTD) for Disease-Free Survival restricted to 60 months at different time horizons for the no pulmonary complication vs. pulmonary complication comparison.

| **Time Horizon** | **No. Trials** | **RMSTD (mos)** | **SE** | **95% CI** | ***p value*** |
| --- | --- | --- | --- | --- | --- |
| 6-month | 3 | 0.3 | 0.1 | 0-0.5 | 0.06 |
| 12-month | 3 | 0.7 | 0.4 | -0.2-1.5 | 0.12 |
| 24-month | 3 | 2.1 | 0.9 | 0.3-3.9 | 0.02 |
| 36-month | 3 | 3.4 | 1.1 | 1.3-5.5 | 0.001 |
| 48-month | 3 | 4.6 | 1.3 | 2-7.2 | <0.001 |
| 60-month | 3 | 5.4 | 1.9 | 1.6-9.1 | 0.005 |

**Legend 3.** SE standard error; 95% CI confidence intervals; mos months.
